# Special Issue: “Feature Papers in Materials Simulation and Design”

**DOI:** 10.3390/ma16051900

**Published:** 2023-02-24

**Authors:** Michele Bacciocchi, Abbas S. Milani

**Affiliations:** 1 Dipartimento di Economia, Scienze e Diritto (DESD), University of San Marino, Via Consiglio dei Sessanta, 47891 Dogana, San Marino; 2 School of Engineering, The University of British Columbia, Kelowna, BC V1V 1V7, Canada

The title of the current Special Issue, “Feature Papers in Materials Simulation and Design”, has identified the aims of this collection since its opening: the gathering of research works and comprehensive review papers that advance the understanding and prediction of material behavior at different scales, from atomistic to macroscopic, through innovative modeling and simulation.

In this context, interdisciplinary researches that tackled challenging and complex material problems where the governing phenomena might span different scales of material behavior, with an emphasis on the development of quantitative approaches to explain and predict experimental observations, have been collected. Likewise, peculiar attention has been given to homogenization techniques for the evaluation of the mechanical properties of new materials and multi-phase composites. Innovative numerical approaches for the mechanical analysis, highlighting their accuracy, reliability, and stability features, have been also welcomed. Significant space has been also given to advanced and sustainable technologies. From the aforementioned topics, it is evident that this Special Issue has represented the ideal forum for disseminating excellent research findings, as well as sharing innovative ideas in this significant field.

Throughout the several months of activity, this Special Issues has been able to attract many interesting researches. Its success is proven by the sixteen papers collected and published, which have passed through the rigorous review process carried out by experts in the field, who should be gratefully acknowledged for their efforts. An heartfelt thanks should be also given to all the authors, coming from many different countries of the world, who contributed to the success of the collection. This important achievement could be barely reached without the constant and kind support given by the Section Managing Editor, Ms. Fay Liu, who should be gratefully thanked for her dedication and commitment. Finally, the Editors-in-Chief of Materials should be also mentioned for the fantastic opportunity of managing this successful Special Issue.

A brief review of the papers published in the collection is now presented as a proof of the advancements and innovations achieved in the field of materials simulation and design. Nurek et al. [1] investigated the possibility of using forest logging residues as an alternative to plant biomass of various origins. In particular, they concluded that shredded logging residues can be used to produce briquettes. A series of test has been presented to discuss the density and compaction of the briquette, providing useful results for manufacturers.

The paper by Tulska et al. [2] has been focused on the kinematics of cone opening in the European larch (*Larix decidua Mill*.) during a four-step seed extraction process and to determine optimum process time on that basis, describing also the microscopic cellular structure of scales in cones for different values of moisture content. Their results have highlighted the conditions for the automation of this process.

De Schryver et al. [3] proved that numerical finite volume simulations are a powerful means in the context of the rheological assessment of concrete. Their results should be used by engineers to build confidence on the reliability of numerical simulations. In addition, they stated that to improve bias due to uncertain rheology, a rheological configuration close to the engineer’s aimed application should be taken into account without overlooking important phenomena.

The paper by Chen et al. [4] aimed at studying the thermal properties and thermoelectric performance of imidazole-graphyne (ID-GY) by combining first principle calculations with the Boltzmann transport theory. A detailed analysis of the harmonic and anharmonic properties has been presented to prove that the low lattice thermal conductivity can be attributed to the low Young’s modulus, low Debye temperature, and high Grüneisen parameter. Finally, the authors demonstrated that it is possible to reduce the lattice thermal conductivity and enhance the thermoelectric performance of carbon-based materials by changing structural units from hexagonal to pentagonal

On the other hand, the paper by Ursini and Collini [5] has been focused on the Fused Deposition Modeling (FDM) additive technology, emphasizing that the analysis of the functional behavior of FDM parts is still a topic of great interest. They investigated experimentally and numerically the effects of different phenomena that could affect the mechanical features of 3D printed lattice structures.

Bragov et al. [6] have discussed the influence of specimen geometry and loading conditions on the mechanical properties of porous brittle media. Their results proved that the structure of the material should be taken into account so that the size of the specimen (in terms of both length and diameter) exceeds the size of the internal fractions of the material by at least five times, when the geometry of specimens of brittle porous media is defined.

A fully autonomous computational workflow to identify light-harvesting materials for water splitting devices based on properties has been presented by Ludvigsen et al. [7]. In particular, they discussed relevant features such as stability, size of the band gap, position of the band edges, and ferroelectricity, proving that ferroelectric materials represents a possible solution to enhance the efficiency of solar cells and photoelectrocatalytic devices in light-harvesting applications.

Chomka et al. [8] investigated, instead, the possibility of using a high-pressure water-ice jet as a new method to remove a worn-out paint coating from the surface of metal parts (including those found in means of transportation) and to prepare the base surface for the application of renovation paint coating. The results have been supported by an experimental campaing.

Vahed et al. [9] highlighted the need of mathematical predictive models to overcome the complex and non-linear nature of material properties evolution during 3D printing, especially when Fused Deposition Modeling (FDM) is considered. Once the process parameters have been discussed, artificial neural networks have been trained to predict both the storage and loss moduli of the samples. An optimization of the process parameters through the Particle Swarm Optimization (PSO) has been also performed.

The topic of green composites has been discussed in the paper by Jiao-Wang et al. [10], developing fully biodegradable composites made of microbially degradable polymers reinforced with natural fibers. In particular, they enhanced numerical models to predict the damage of these structures with the aim to extend their employment in industrial applications of structural responsibility.

Civalek et al. [11] developed an efficient eigenvalue algorithm for the axial vibration analysis of embedded short-fiber-reinforced micro-/nano-composite rods subjected to arbitrary boundary conditions. Their investigations have been carried out within the framework provided by the nonlocal elasticity theory needed to capture the size effect. The influence of elastic spring boundary conditions on the axial vibration frequencies and mode shapes has been also discussed.

A beam model for thermal buckling analysis of a bimetallic box beam has been developed by Simonetti et al. [12], considering Euler–Bernoulli–Vlasov beam theory and including large rotations but small strains. An updated Lagrangian formulation is used to discuss the nonlinear stability analysis. The effects of different boundary conditions, beam lengths and material thickness ratios on the critical buckling temperature and post-buckling responses have been discussed through several numerical tests.

The results presented in the paper by Esencan Türkaslan et al. [13] proved that the efficiency of the photocatalytic activity of the nanocomposite depends on different synthesis methods and the morphology resulting from the changed method. To this aim, tungsten trioxide/graphene oxide nanocomposites have been successfully synthesized using in situ and ex situ chemical approaches.

A series of experimental and numerical studies concerning the influence of temperature on mode II fracture of a T800/epoxy unidirectional laminates has been presented by Gong et al. [14]. The failure mechanism has been also determined by using a scanning electron microscope. The validity of the numerical model has been confirmed by the experimental tests. Therefore, the results represent an helpful guidance for the design of composite laminates.

Dastjerdi et al. [15] developed a novel nonlinear elasticity theory for annular and circular plates made of hyperelastic materials. The effect of viscosity has been taken into account to obtain the long-term structural response. The partial differential equations have been solved through a semi-analytical method, and have been confirmed by the successful comparison with the results found in other sources.

The last paper by Cheng and Vescovini [16] presented instead an accurate and efficient numerical method for the analysis and design of Variable Stiffness (VS) laminates. In particular, a ps-version of the Finite Elements Method (ps-FEM) has been developed for the global/local analysis through different refinement approaches. Peculiar attention has been given to the implementation aspects to illustrate the potential of the methodology.

The Guest Editors would like to congratulate all the authors for the remarkable results presented in these papers.
